# Disorganization in first episode schizophrenia: psychopathological findings and treatment response from a 2-year Italian follow-up research in a real-world setting

**DOI:** 10.1192/j.eurpsy.2023.960

**Published:** 2023-07-19

**Authors:** L. Pelizza, E. Leuci, E. Quattrone

**Affiliations:** Department of Mental Health, AUSL di Parma, Parma, Italy

## Abstract

**Introduction:**

Disorganization is a core dimension of schizophrenia, yet it is relatively under-investigated compared to positive and negative ones, especially at the illness onset. Indeed, most of the empirical studies investigating the disorganized domain included patients with prolonged schizophrenia.

**Objectives:**

Thus, the aims of this research were (1) to monitor the longitudinal stability of disorganized symptoms in young patients with First Episode Schizophrenia (FES) along a 2-year follow-up period, and (2) to examine any significant association of disorganization with functioning, psychopathology and the specific treatment components of an “Early Intervention in Psychosis” (EIP) program across the 2 years of follow-up.

**Methods:**

At baseline, 159 FES individuals (aged 12–35 years) completed the Positive And Negative Syndrome Scale (PANSS) and the Global Assessment of Functioning (GAF). Spearman’s correlation coefficients and multiple linear regression analysis were carried out. Specifically, the PANSS “Disorganization” dimension included 8 PANSS items: “Conceptual Disorganization”, “Difficulty in Abstract Thinking”, “Stereotyped Thinking”, “Mannerisms and Posturing”, “Disorientation”, “Poor Attention”, “Disturbance of Volition” and “Preoccupation”.

**Results:**

At baseline, the PANSS “Disorganization” dimension score had significant positive correlations with all other PANSS factor subscores, as well as significant negative correlation with the GAF score. The statistically strongest association was with the PANSS “Negative” domain score. Along the 2-year follow-up period, a significant decrease in the PANSS “Disorganization” dimension subscore was observed. This reduction was related to score decreases in all the other PANSS domains (especially the “Negative” one) and an increase in GAF scores. Furthermore, decreaes in PANSS “Disorganization” dimension scores showed a significant positive correlation with the total number of individual psychotherapy sessions provided to FES patients during the first year of the Pr-EP protocol (also confimed by our multiple linear regression analysis results).

**Conclusions:**

Disorganization is clinically relevant in FES patients, already at the recruitment within specialized EIP programs. In particular, disorganized dimension in FES had significant enduring associations with functioning deterioration and negative symptoms. However, improvement in disorganization levels seems to be due to the intensity of individual psychotherapy sessions offered to FES individuals in the first year of intervention within specific EIP programs.

**Disclosure of Interest:**

None Declared

